# Vitamin A enhanced periosteal osteoclastogenesis is associated with increased number of tissue-derived macrophages/osteoclast progenitors

**DOI:** 10.1016/j.jbc.2024.107308

**Published:** 2024-04-23

**Authors:** Petra Henning, Anna Westerlund, Karin Horkeby, Vikte Lionikaite, Karin H. Nilsson, Sofia Movérare-Skrtic, H. Herschel Conaway, Ulf H. Lerner

**Affiliations:** 1 Department of Internal Medicine and Clinical Nutrition, Sahlgrenska Osteoporosis Centre and Centre for Bone and Arthritis Research, Institute for Medicine, Sahlgrenska Academy at University of Gothenburg, Gothenburg, Sweden; 2 Department of Physiology and Cell Biology, University of Arkansas for Medical Sciences, Little Rock, Arkansas, USA

**Keywords:** vitamin A, osteoclasts, osteoblasts, periosteum, macrophages

## Abstract

A deleterious effect of elevated levels of vitamin A on bone health has been reported in clinical studies. Mechanistic studies in rodents have shown that numbers of periosteal osteoclasts are increased, while endocortical osteoclasts are simultaneously decreased by vitamin A treatment. The present study investigated the *in vitro* and *in vivo* effect of all-*trans* retinoic acid (ATRA), the active metabolite of vitamin A, on periosteal osteoclast progenitors. Mouse calvarial bone cells were cultured in media containing ATRA, with or without the osteoclastogenic cytokine receptor activator of nuclear factor kappa B-ligand (RANKL), on plastic dishes or bone discs. Whereas ATRA did not stimulate osteoclast formation alone, the compound robustly potentiated the formation of RANKL-induced bone resorbing osteoclasts. This effect was due to stimulation by ATRA (half-maximal stimulation ∼3 nM) on the numbers of macrophages/osteoclast progenitors in the bone cell cultures, as assessed by mRNA and protein expression of several macrophage and osteoclast progenitor cell markers, such as macrophage colony-stimulating factor receptor, receptor activator of nuclear factor kappa B, F4/80, and CD11b, as well as by flow cytometry (FACS) analysis of CD11b^+^/F480^+^/Gr1^-^ cells. The stimulation of macrophage numbers in the periosteal cell cultures was not mediated by increased macrophage colony-stimulating factor or interleukin-34. In contrast, ATRA did not enhance macrophages in bone marrow cell cultures. Importantly, ATRA treatment upregulated the mRNA expression of several macrophage-related genes in the periosteum of tibia in adult mice. These observations demonstrate a novel mechanism by which vitamin A enhances osteoclast formation specifically on periosteal surfaces.

Vitamin A, a lipid-soluble micronutrient consumed in the diet, is the only known compound able to induce spontaneous fractures in the long bones of animals (1). More importantly, increased vitamin A (retinol) intake in humans has been associated with decreased bone mineral density and increased bone fragility (2, 3, 4, 5). Elevated serum retinol levels have also been linked to increased risk of hip fractures (4, 6, 7). However, not all investigations have been able to reproduce these associations and in some studies no associations between vitamin A intake or serum retinol levels and bone mineral density or increased fracture risk have been observed (reviewed in (8, 9, 10)). In two meta-analyses using data from three and five studies, respectively, increased serum retinol was found to be associated with increased risk for hip fractures in one study (6) but not in the other (11).

*De novo* synthesis of vitamin A is not possible and, therefore, it must be consumed in the diet. Dietary vitamin A is obtained as either retinyl esters or ß-carotenoids. Retinyl esters and ß-carotene are transported in chylomicrons to the liver, where they are either stored as retinyl esters or converted to retinol and released into the blood as a complex with retinol-binding protein 4 and transthyretin. In target cells, retinol is oxidized in two steps to the active metabolite all-*trans* retinoic acid (ATRA), which is translocated to the nucleus where ATRA binds to retinoic acid receptors (RARs), of which there are three subtypes: α, β, γ. ATRA-induced cellular changes occur through the heterodimerization of RARs and retinoic X receptors, which activates retinoic acid response elements in the promoter regions of target genes and thereby regulates gene transcription (reviewed in (8, 9, 12, 13, 14, 15, 16)).

The induction of hypervitaminosis A in rodents for short durations (∼7 days) have illustrated that toxic doses of vitamin A increase the number of bone resorbing osteoclasts located on periosteal bone surfaces (17, 18, 19), while the numbers of endocortical osteoclasts are decreased (18, 19), resulting in reduced cortical bone thickness and bone strength (17, 20). More recently, we showed that vitamin A for longer duration (4–10 weeks), at more clinically relevant doses, is also able to reduce cortical bone mass (19). These observations, together with human data linking elevated vitamin A levels to increased risk of fractures (2, 3, 4, 6, 7), illustrates the negative effects of excess vitamin A on bone metabolism.

Osteoclast progenitor cells originate from myeloid hematopoietic stem cells and are closely related to monocytes/macrophages and dendritic cells in the immune system (21). Proliferation and survival of mononuclear osteoclast precursor cells is dependent on macrophage colony-stimulating factor (M-CSF)/colony-stimulating factor 1 (CSF1) (22). Interestingly, interleukin-34 (IL-34) is also able to bind to the receptor for M-CSF and can substitute for M-CSF during osteoclast differentiation (23). Differentiation of osteoclasts is driven by binding of receptor activator of nuclear factor kappa B-ligand (RANKL), produced in bone tissue by late osteoblasts, osteocytes and bone marrow stromal cells (24, 25, 26), to its membrane receptor activator of nuclear factor kappa B (RANK) on osteoclast progenitors, an interaction which can be inhibited by osteoprotegerin (OPG) (27). Signaling events downstream RANK receptor includes activation of mitogen-activated protein kinase and transcription factors, such as nuclear factor κB, c-Fos-containing activator protein-1, and the master regulator of osteoclastogenesis nuclear factor of activated T cells, cytoplasmic 1 (NFATc1) (28). RANK signaling also induces transcriptional repression of antiosteoclastogenic transcription factors expressed by macrophages, such as interferon regulatory factor 8 (Irf8) and MAF BZIP transcription factor B (29). Important for the formation of mature osteoclasts is the induction of genes involved in osteoclast activity such as *Acp5* (encoding tartrate resistant acid phosphatase; TRAP), encoding cathepsin K (*Ctsk*), and encoding calcitonin receptor (*Calcr*).

In addition to human studies and *in vivo* animal studies, *ex vivo* bone organ cultures of mouse calvaria and rat tibia have demonstrated that vitamin A indeed possesses the ability to increase osteoclast formation and bone resorption (30, 31, 32, 33, 34, 35, 36) due to an increase in RANKL (37). This provides further evidence that vitamin A increases osteoclastogenesis and bone resorption and that excess vitamin A may be a risk factor for secondary osteoporosis.

In contrast to *in vivo* and *ex vivo* organ culture experiments, *in vitro* studies using osteoclast progenitors isolated from mouse bone marrow and human blood have demonstrated that vitamin A inhibits osteoclast formation. ATRA has been shown to inhibit physiologically induced (RANKL, parathyroid hormone and 1,25(OH)_2_-vitamin D3) and inflammatory-induced (tumor necrosis factor-α, lipopolysaccharide) osteoclastogenesis in cultures of bone marrow macrophages from rats and mice (17, 38, 39, 40, 41, 42, 43) and in human blood CD14^+^ monocyte cultures (43, 44, 45). This inhibition has been attributed to activation of RARα (38, 42, 43) and RARγ (41). The *in vitro* observations reporting inhibitory effects by vitamin A on osteoclastogenesis may be reconciled with *in vivo* findings of decreased endocortical osteoclasts, since cells used for these experiments are often isolated from bone marrow adjacent to endocortical bone.

The contradictory osteoclastogenic response to vitamin A *in vivo/ex vivo* and *in vitro*, as well as the *in vivo* site-specific responses, highlights the heterogeneity of osteoclastogenesis (46). Thus far, no explanation exists as to why vitamin A has these opposing effects. Therefore, the aim of the present study was to investigate how osteoclast progenitors present in periosteal tissues respond to vitamin A with the hypothesis that these cells may respond differently than the bone marrow cells (BMCs).

## Results

### ATRA alone does not stimulate osteoclast formation in periosteal bone cell cultures

**Figure 1:**
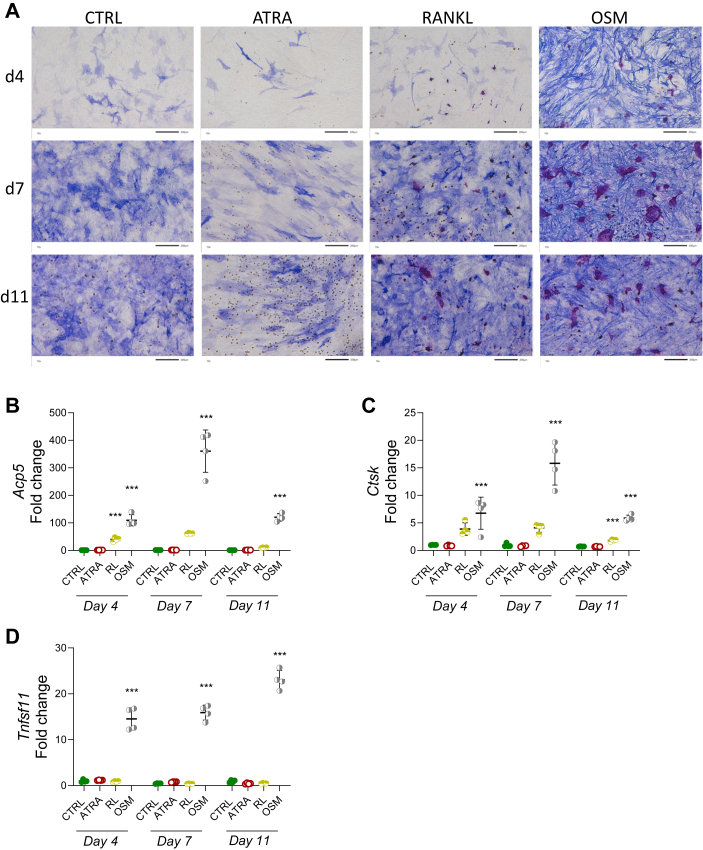
**ATRA alone did not stimulate osteoclast formation in periosteal bone cell cultures.** Cultures of periosteal bone cells were treated with control (CTRL), ATRA (100 nM), RANKL (RL; 4 ng/ml), or oncostatin M (OSM; 100 ng/ml). Alkaline phosphatase and TRAP-stained images (*A*) of culture at day 4, 7, and 11, the scale bars represent 200 μm. The mRNA expression of *Acp5* (*Trap*) (*B*), *Ctsk* (*C*), and *Tnfsf11* (*Rankl*) (*D*). Figures are displayed as *scatter plots* of replicate wells with mean ± SD, one-way ANOVA followed by Dunnett’s multiple comparison test *versus* control at each time point. ∗∗∗*p* < 0.001. ATRA, all-*trans* retinoic acid; *Ctsk*, cathepsin K; RANKL, receptor activator of nuclear factor kappa B-ligand; TRAP, tartrate resistant acid phosphatase.

We used primary mouse periosteal bone cell cultures containing both osteoblast and osteoclast progenitors to investigate the effect of ATRA on osteoclastogenesis (47, 48). The addition of ATRA was compared to the effect of RANKL, which induces osteoclast differentiation by acting directly on osteoclast progenitors. Oncostatin M (OSM), a cytokine in the IL-6 family which is a robust stimulator of osteoclast formation *in vivo* (49) and *in vitro* (50), was used as a positive control for agents inducing osteoclast formation indirectly by increasing the RANKL/OPG ratio expression in osteoblasts. While the addition of RANKL and OSM generated many TRAP-positive mononucleated cells as well as multinucleated TRAP-positive osteoclasts, no TRAP-positive cells could be seen in cultures stimulated with only ATRA (Fig. 1*A*). The expression of the osteoclastic genes *Acp5* (encoding TRAP) and *Ctsk* (encoding cathepsin K) was induced by both RANKL and OSM, but unchanged by ATRA (Fig. 1, *B* and *C*). While OSM strongly induced *Tnfsf11* (encoding RANKL) mRNA expression, the addition of ATRA did not (Fig. 1*D*). The periosteal cell cultures were also stained for alkaline phosphatase, demonstrating the presence of osteoblasts in the cultures (Fig. 1*A*). It is apparent that ATRA decreased the numbers of alkaline phosphatase positive cells, whereas OSM enhanced their numbers, and that RANKL had no obvious effect.

### Osteoclast differentiation and bone resorption are enhanced by ATRA in prolonged RANKL-stimulated periosteal bone cell cultures

**Figure 2:**
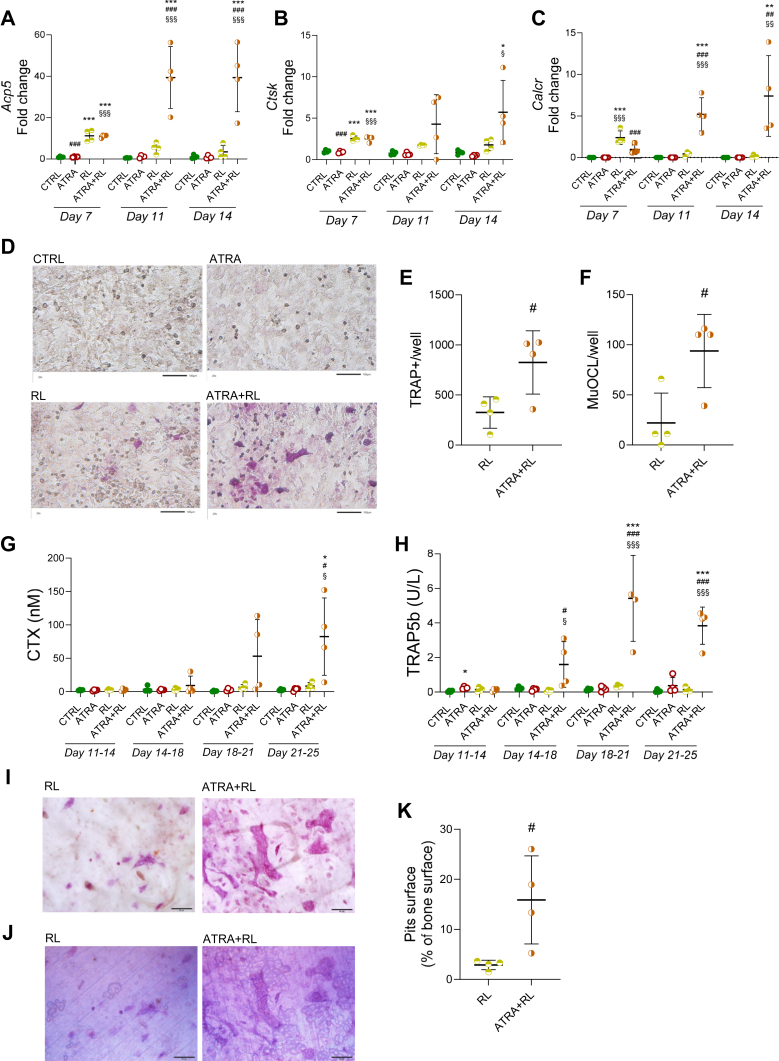
**ATRA enhanced osteoclast differentiation and bone resorption in prolonged RANKL-stimulated periosteal bone cell cultures.** Periosteal cells cultured on plastic dishes and bone discs in control (CTRL), ATRA (100 nM), RANKL (RL; 4 ng/ml), or RANKL + ATRA-containing media. Gene expression of *Acp5* (*Trap*) (*A*), *Ctsk* (*B*), and *Calcr* (*C*) after 7, 11, and 14 days of culture on plastic dishes. TRAP-stained images (*D*) and the numbers of TRAP-positive cells (*E*) and multinucleated TRAP-positive osteoclasts (MuOCL) (*F*) of periosteal cells cultured on plastic for 14 days. The scale bars represent 100 μm. CTX (*G*) and TRAP5b (*H*) measured in media from cultures on bone discs from day 11 to 14, 14 to 18, 18 to 21, and 21 to 25. TRAP-stained images (*I*) and reflective light images (*J*) of cells cultured on bone discs for 25 days. The scale bars represents 20 μm. Quantification (*K*) of pits surface per bone surface after 25 days culture. Figures are displayed as *scatter plots* of replicate wells with mean ± SD. *A*, *B*, *C*, *G*, and *H*, one-way ANOVA followed by Tukey’s multiple comparison test at each time point. *E*, *F*, and *K*, Students unpaired, two-tailed *t* test; ∗*p* < 0.05, ∗∗*p* < 0.01, and ∗∗∗*p* < 0.001 *versus* control. ^§^*p* < 0.05, ^§§^*p* < 0.01, and ^§§§^*p* < 0.001 *versus* ATRA. ^#^*p* < 0.05, ^##^*p* < 0.01, and ^###^*p* < 0.001 *versus* RANKL. ATRA, all-*trans* retinoic acid; *Calcr*, calcitonin receptor; *Ctsk*, cathepsin K; CTX, C-terminal telopeptides of type I collagen; RANKL, receptor activator of nuclear factor kappa B-ligand; TRAP, tartrate resistant acid phosphatase.

To further study the effect of ATRA on mouse calvarial periosteal osteoclast progenitors, the effect of ATRA on osteoclastogenesis stimulated by RANKL in periosteal bone cell cultures was investigated. The expression of the osteoclastic genes *Acp5, Ctsk*, and the highly osteoclast specific *Calcr* were observed after 7, 11, and 14 days of culture in RANKL-containing medium with or without ATRA (Fig. 2, *A*–*C*). All three osteoclastic genes were induced by RANKL after 7 days. Cotreatment with ATRA did not affect expression of *Acp5*, *Ctsk*, or *Calcr* after 7 days. Interestingly, however, in prolonged cultures (11 and 14 days) the combination of ATRA and RANKL resulted in significantly higher expression of osteoclastic genes than RANKL alone.

TRAP-staining after 14 days of culture on plastic dishes showed 2.5-fold higher numbers of TRAP^+^ cells and 4-fold higher numbers of multinucleated osteoclast (MuOCL) in RANKL + ATRA-treated cell cultures than the treatment with RANKL alone (Fig. 2, *D*–*F*).

To study if the increased osteoclastogenesis was associated with increased resorption, the periosteal bone cells were cultured on bone discs in the presence of ATRA, RANKL, or the combination of RANKL and ATRA for 25 days. Bone resorption, analyzed by C-terminal telopeptides of type I collagen (CTX) released from the bone discs, was not affected by the addition of ATRA alone but was robustly enhanced by the addition of ATRA to RANKL-stimulated cells (Fig. 2G). Osteoclast formation, assessed by quantifying TRAP5b in the culture media, also showed that the combination of RANKL and ATRA resulted in greatly enhanced osteoclastogenesis (Fig. 2*H*). TRAP staining at the end of the cultures and analysis of the surface of excavation pits confirmed that ATRA robustly enhanced formation of bone resorbing osteoclasts (Fig. 2, *I*–*K*) as compared to findings in cell cultures stimulated with RANKL alone.

### Priming of the periosteal cells with ATRA enhances RANKL-induced osteoclast formation

**Figure 3:**
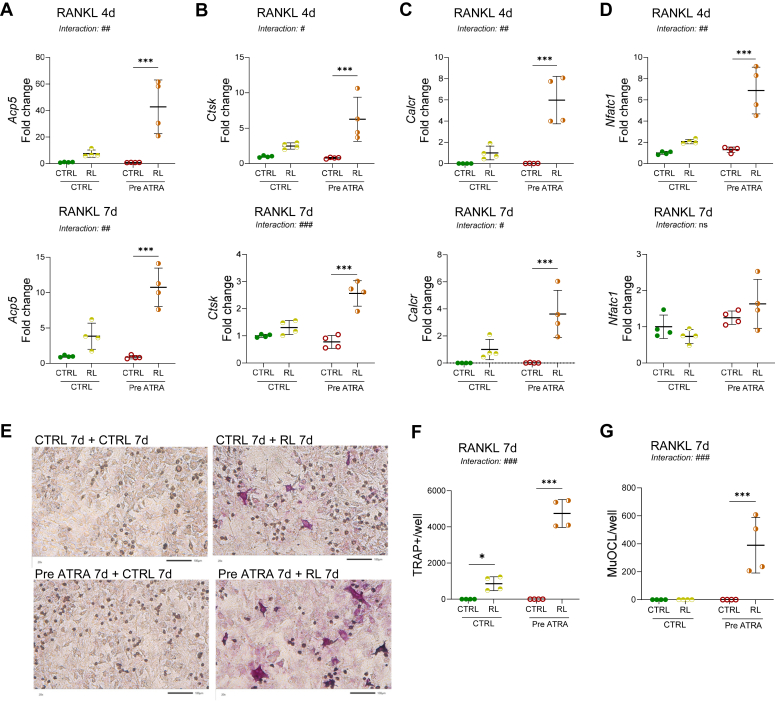
**Pretreatment with ATRA increased the number of RANKL-induced TRAP-positive cells and osteoclasts.** Periosteal bone cell cultures were preincubated with or without ATRA (100 nM) for 7 days. Thereafter, ATRA or control media was replaced by new control or RANKL-containing media (4 ng/ml). Gene expression of *Acp5* (*A*), *Ctsk* (*B*), *Calcr* (*C*), and *Nfatc1* (*D*) was analyzed 4 and 7 days after addition of RANKL. TRAP staining (*E*) and counting of TRAP-positive cells (*F*) and multinucleated TRAP positive osteoclast (MuOCL) (*G*) was performed after 7 days of RANKL treatment. The scale bars represents 100 μm. Figures are displayed as *scatter plots* of replicate wells with mean ± SD. Two-way ANOVA followed by Sidak’s multiple comparison test for the effect of RANKL with or without pretreatment with ATRA; ∗*p* < 0.05, ∗∗*p* < 0.01, ∗∗∗*p* < 0.001 *versus* CTRL; interaction ^#^*p* < 0.05, ^##^*p* < 0.01, ^###^*p* < 0.001 for the difference in RANKL response in cells pretreated in CTRL *versus* ATRA-containing media. ATRA, all-*trans* retinoic acid; *Calcr*, calcitonin receptor; *Ctsk*, cathepsin K; ns, nonsignificant; RANKL, receptor activator of nuclear factor kappa B-ligand; TRAP, tartrate resistant acid phosphatase.

To investigate if priming of the periosteal cells with ATRA enhances the response to RANKL, calvarial bone cells were precultured with or without ATRA for 7 days, after which ATRA was removed and cells were further cultured with or without RANKL for 4 or 7 days to induce osteoclast differentiation. In cells pretreated with ATRA, RANKL stimulation for 4 or 7 days induced a significantly increased mRNA expression of *Acp5*, *Ctsk*, and *Calcr* (Fig. 3, *A*–*C*) compared with the expression observed when cells had been precultured in control medium for 4 or 7 days. The mRNA expression of the osteoclastogenic transcription factor *Nfatc1* was increased after 4 days, but not after 7 days of RANKL treatment in ATRA-precultured cells (Fig. 3*D*).

Preculture in ATRA for 7 days resulted in a robust increase in the number of TRAP-positive mononuclear cells and multinucleated osteoclasts after subsequent RANKL treatment for 7 days (Fig. 3, *E*–*G*).

These findings indicate that ATRA increases the numbers of osteoclast progenitors and/or their osteoclastogenic potential.

### ATRA increases the phenotypic markers of osteoclast progenitors and macrophages in periosteal bone cell cultures

**Figure 4:**
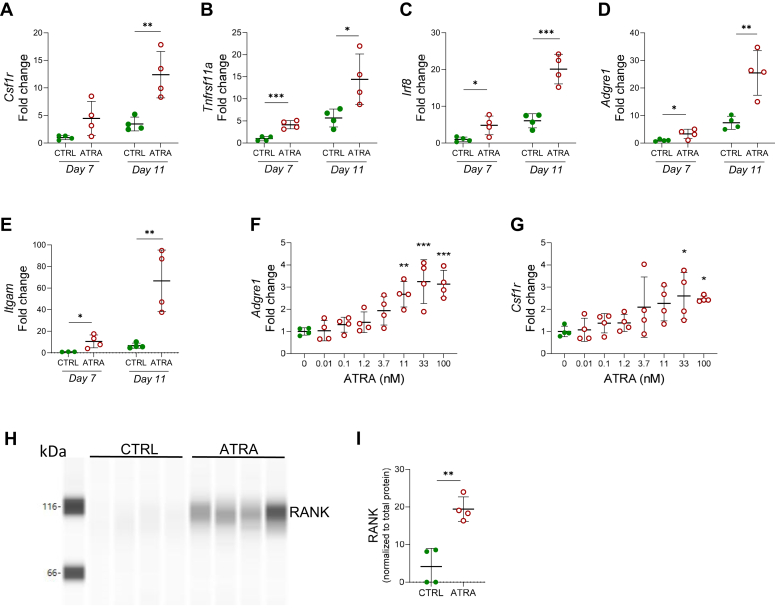
**ATRA increased the expression of macrophage and osteoclast progenitor-related genes and Rank protein in periosteal bone cell cultures.** The mRNA expression of *Csf1r (mcsf-r)* (*A*), *Tnfrsf11a* (*Rank*) (*B*), *Irf8* (*C*)*, Adgre1 (F4/80)* (*D*), and *Itgam* (*CD11b*) (*E*) in periosteal bone cells cultured in control (CTRL) or ATRA-containing media(100 nM) for 7 and 11 days. The mRNA expression of *Adgre1 (F4/80)* (*F*) and *Csf1r (mcsf-r)* (*G*) in periosteal bone cell cultured in control (CTRL) media or with different doses of ATRA (0.01–100 nM) for 11 days. Lane view (*H*) and quantification (*I*) of RANK protein levels analyzed by capillary-based electrophoresis immunodetection (Simple Western) in cultures treated with CTRL or ATRA-containing media for 11 days (n = 4 replicate wells). Figures are displayed as scatter plots of replicate wells with mean ± SD. *A*, *B*, *C*, *D*, *E*, and *I*, unpaired, two-tailed Student’s *t* test at each time point, *F* and *G*, one-way ANOVA followed by Dunnett’s multiple comparison test *versus* control. ∗*p* < 0.05, ∗∗*p* < 0.01, and ∗∗∗*p* < 0.001. ATRA, all-*trans* retinoic acid; *Csf1r*, CSF1 receptor; CSF1, colony-stimulating factor 1; M-CSF, macrophage colony-stimulating factor; *Irf8*, interferon regulatory factor 8; RANK, receptor activator of nuclear factor kappa B.

The mRNA expression of *Csf1r* (encoding the CSF1/M-CSF receptor) and *Tnfrsf11a* (encoding RANK) was induced by ATRA after treatment of the periosteal cells for 7 or 11 days (Fig. 4, *A* and *B*). This may reflect either a higher number of M-CSF– and RANKL-responsive cells in the cultures or increased expression of these transcripts in individual progenitors, making the cells more responsive to M-CSF and RANKL. Interestingly, the mRNA expression of macrophage markers such as *Irf8* (encoding interferon regulatory factor 8)*, Adgre1* (encoding F4/80), and *Itgam* (encoding CD11b) was also induced by ATRA, indicating the presence of enhanced numbers of macrophages/osteoclast precursors in response to ATRA (Fig. 4, *C*–*E*). The stimulation of *Adgre1* and *Csf1r* mRNA was dependent on the concentration of ATRA with effects observed at 0.1 nM and above and with half-maximal stimulation equaling ∼3 nM (Fig. 4, *F* and *G*).

ATRA treatment for 11 days increased also protein expression of RANK (Fig. 4, *H* and *I*).

### ATRA increases the number of macrophages in periosteal bone cell cultures

To obtain conclusive evidence for a stimulatory effect by ATRA on the number of macrophages/osteoclast progenitor cells in the periosteal cells, rather than enhancing the mRNA and protein expression of macrophage/osteoclast progenitor cell markers in individual cells, we performed FACS analyses.

**Figure 5:**
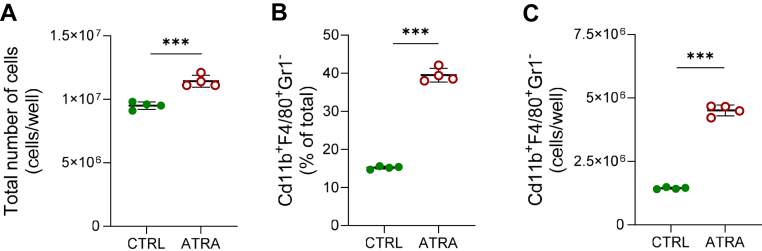
**ATRA increased the number of macrophages/osteoclast progenitors in periosteal bone cell cultures.** Total number of cells/well (*A*) and percentage (*B*) and number (*C*) of CD11b^+^F4/80^+^Gr1^−^ cells after 11 days culture in control (CTRL) or ATRA-containing media (100 nM). Figures are displayed as scatter plots of replicate wells with mean ± SD. Unpaired, two-tailed Student’s *t* test, ∗∗∗*p* < 0.001. ATRA, all-*trans* retinoic acid.

We demonstrated that the total numbers of cells in the cultures were higher after treatment with ATRA for 11 days than controls (Fig. 5*A*). Interestingly, 15.2 ± 0.2% of the cells were CD11b^+^F4/80^+^Gr1^−^ in control cultures and 39.5 ± 0.9% in cultures treated with ATRA (Fig. 5*B*). ATRA caused a 3-fold increase of the number of CD11b^+^F4/80^+^Gr1^−^ cells (Fig. 5*C*). Increased numbers of CD11b^+^F4/80^+^Gr1^-^ cells after treatment with ATRA have been observed in five independent experiments.

Since the mRNA expression of *Tnfsf11* and *Tnfrsf11b* did not differ in RANKL-stimulated cells precultured in control medium or with ATRA (Fig. S1), these data show that the ATRA-induced enhanced osteoclastogenic response to RANKL and increased expression of macrophage transcripts is due to an increase of the numbers of macrophages/osteoclast progenitors independent of increased production of RANKL and/or decreased OPG.

### ATRA does not increase the expression of macrophage transcripts in mouse BMC cultures

**Figure 6:**
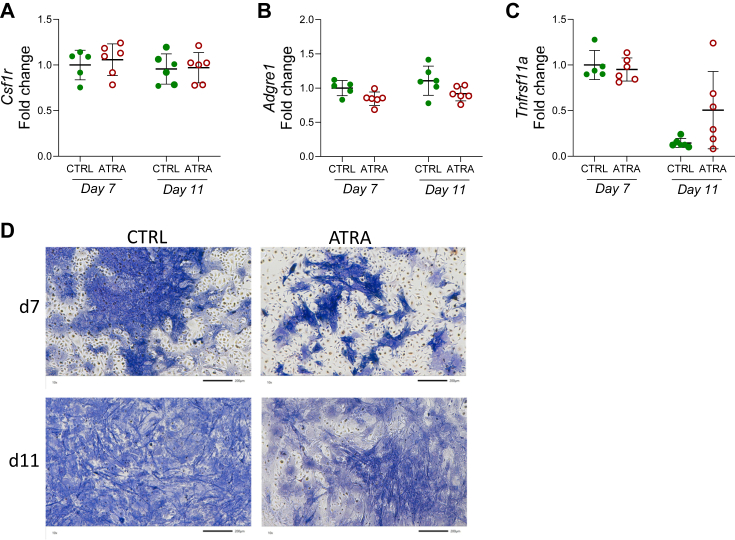
**ATRA did not alter the expression of macrophage and osteoclast progenitor-related genes in whole bone marrow cell cultures.** The mRNA expression of *Csf1r* (*A*), *Adgre1 (F4/80)* (*B*), and *Tnfrsf11a* (*Rank*) (*C*) in bone marrow cells cultured in control (CTRL) or ATRA-containing media (100 nM) for 7 or 11 days. Figures are displayed as *scatter* plots of replicate wells with mean ± SD. No significant differences detected by unpaired, two-tailed student’s *t* test at each time point. Alkaline phosphatase and TRAP staining of bone marrow cells cultured in CTRL or ATRA-containing media for 7 or 11 days (*D*). The scale bars represent 200 μm. ATRA, all-*trans* retinoic acid; *Csf1r*, CSF1 receptor; RANK, receptor activator of nuclear factor kappa B; TRAP, tartrate resistant acid phosphatase.

Crude BMC cultures, containing pluripotent stromal cells and hematopoietic cells including macrophages/osteoclast progenitor cells, can be used for studies on osteoclast formation when studying effects by hormones and cytokines which stimulate osteoclast formation by inducing RANKL in stromal cells (51, 52). In BMC cultures, containing macrophages as assessed by the mRNA expression of macrophage genes *Csf1r* and A*dgre1* (Fig. 6, *A* and *B*) as well as alkaline phosphatase positive stromal cells/osteoblasts (Fig. 6*D*), treatment with ATRA for 7 or 11 days did not affect the mRNA expression of *Csf1r*, *Adgre,1* or *Tnfrsf11a* (Fig. 6, *A*–*C*), indicating that the numbers of macrophages are not altered by ATRA in these cultures.

### Enhanced osteoclastogenesis by ATRA is not mediated by M-CSF or IL-34

Since M-CSF is a well-known stimulator of bone marrow macrophages (53), we next investigated if ATRA increased the number of macrophages/osteoclast progenitors in the periosteal cell cultures through increased M-CSF.

**Figure 7:**
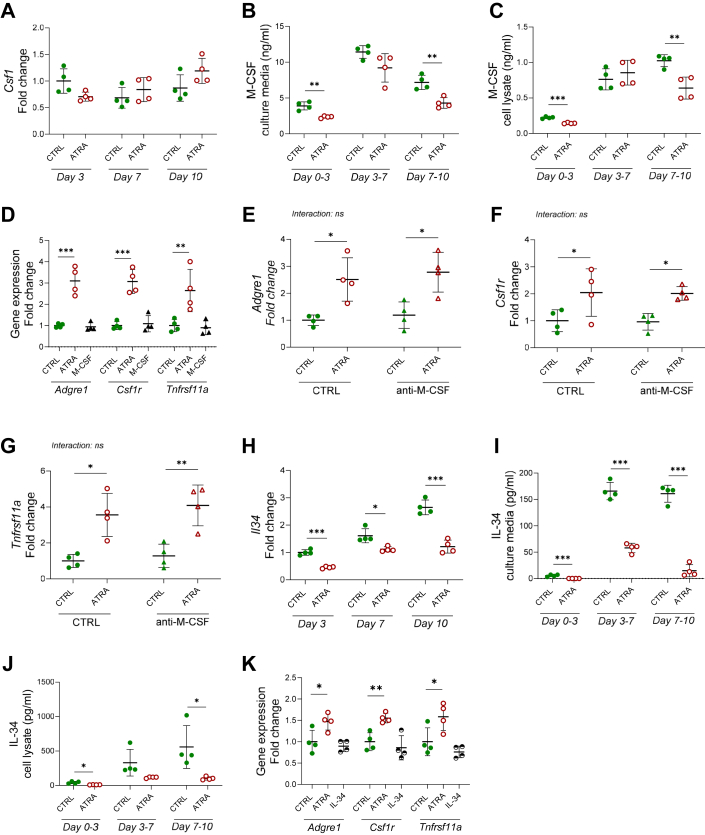
**Enhanced osteoclastogenesis by ATRA is not associated with enhanced CSF1 or IL-34.** Periosteal cells cultured in control (CTRL), ATRA (100 nM), M-CSF (30 ng/ml), and/or anti-M-CSF–containing media (4 μg/ml). The expression of *Csf1 (m-csf)* mRNA (*A*) and Csf1/M-CSF protein in culture media (*B*) and periosteal bone cell lysates (*C*). The expression *of Adgre1 (F4/80), Csf1r (mcsf-r),* and *Tnfrsf11a (Rank)* mRNA in periosteal cell treated with CTRL, ATRA, or M-CSF for 10 days (*D*). The expression *of Adgre1 (F4/80)* (*E*)*, Csf1r (mcsf-r)* (*F*), and *Tnfrsf11a (Rank)* (*G*) mRNA in periosteal cell treated with CTRL, ATRA, anti-M-CSF or anti-M-CSF + ATRA for 10 days. *Il34* mRNA (*H*) and IL34 protein in culture media (*I*) and periosteal bone cell lysates (*J*). The expression *of Adgre1 (F4/80), Csf1r (mcsf-r),* and *Tnfrsf11a (Rank)* mRNA in periosteal cell treated with CTRL, ATRA, or IL-34 for 10 days (*K*). Figures are displayed as *scatter plots* of replicate wells with mean ± SD. *A*, *B*, *C*, *H*, *I*, and *J*, unpaired, two-tailed Student’s *t* test at each time point, (*D* and *K*), one-way ANOVA followed by Dunnett’s multiple comparison test *versus* control for each gene. *E*–*G*, two-way ANOVA followed by Sidak’s multiple comparison test for the effect of ATRA in the presence and absence of anti-M-CSF. ∗*p* < 0.05, ∗∗*p* < 0.01, and ∗∗∗*p* < 0.001. ATRA, all-*trans* retinoic acid; CSF-1, colony-stimulating factor 1; *Csf1r*, CSF1 receptor; IL, interleukin; M-CSF, macrophage colony-stimulating factor; RANK, receptor activator of nuclear factor kappa B.

*Csf1* mRNA expression was not altered by ATRA treatment (Fig. 7*A*), but M-CSF protein level in the periosteal cells and culture media was decreased by ATRA (Fig. 7, *B* and *C*). In contrast to ATRA, M-CSF did not increase the mRNA expression of *Adgre1*, *Csf1r,* and *Tnfrsf11*a after 10 days (Fig. 7*D*). Antiserum neutralizing M-CSF did not affect the basal or ATRA-stimulated expression of *Adgre1*, *Csf1r* and *Tnfrsf11a* after 10 days (Fig. 7, *E*–*G*).

IL-34 also binds to the M-CSF receptor and has been shown to be able to substitute for M-CSF in RANKL-induced osteoclast differentiation *in vitro* (23). IL-34 mRNA and protein expression was observed in the periosteal cell cultures and the expression increased progressively during a 10-day culture period (Fig. 7, *H*–*J*). However, ATRA robustly decreased both the mRNA expression of *Il34* and the amounts of IL-34 protein in cells and culture media (Fig. 7, *H*–*J*). In contrast to ATRA, IL-34 did not enhance the mRNA expression of *Adgre1*, *Csf1r,* or *Tnfrsf11a* (Fig. 7*K*).

These results suggest that the increase of osteoclast progenitors induced by ATRA is not mediated by M-CSF or IL-34.

### Hypervitaminosis A in mice enhances the expression of macrophage/osteoclast progenitor genes in tibia periosteum

**Figure 8:**
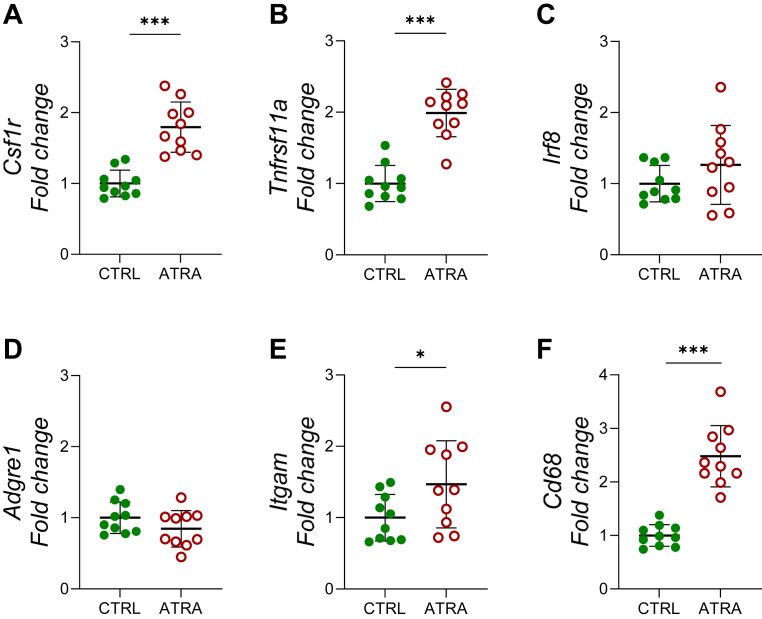
**Oral treatment with ATRA increased the expression of macrophage and osteoclast progenitor related genes in periosteum *in vivo*.** Mice were treated with ATRA (110 mg/kg/day) orally for three consecutive days and gene expression in tibia periosteum was analysed the day after the last treatment. The mRNA expression of *Csf1r (mcsf-r)* (*A*), *Tnfrsf11a* (*Rank*) (*B*), *Irf8* (*C*)*, Adgre1 (F4/80)* (*D*)*, Itgam* (*CD11b*) (*E*), and *CD68* (*F*) in the periosteum from ATRA and vehicle-treated mice. Figures are displayed as *scatter plots* of individual mice with mean ± SD. Unpaired, two-tailed Student’s *t* test. ∗*p* < 0.05, ∗∗∗*p* < 0.001. ATRA, all-*trans* retinoic acid; *Csf1r*, CSF1 receptor; *Irf8*, interferon regulatory factor 8; M-CSF, macrophage colony-stimulating factor; RANK, receptor activator of nuclear factor kappa B.

Our finding that ATRA enhances macrophage/osteoclast progenitor cells in mouse calvarial bone cell cultures is compatible with the finding that hypervitaminosis A increases osteoclast formation on endocranial surfaces of calvarial bones (54). Since a well-known sequala of hypervitaminosis A is decreased cortical bone mass in long bones through enhanced osteoclast formation on periosteal surfaces (17, 18, 19), we treated adult mice with ATRA (tretinoin) orally for 3 days and examined the expression of macrophage/osteoclast progenitor genes in tibia periosteum 1 day later. Analysis of RNA extracted from intact tibia to preferentially isolate RNA from the periosteum showed that treatment robustly upregulated the mRNA expression of *Csf1r*, *Tnfrsf11a, Itgam,* and *Cd68,* whereas *Adgre1* and *Irf8* mRNA was unaffected (Fig. 8, *A*–*F*).

## Discussion

Clinical studies have shown that increased serum levels of vitamin A (retinol) are associated with decreased bone mineral density and increased fracture susceptibility (4, 8). Rodents given high doses of vitamin A exhibit decreased cortical bone mass due to excessive formation of osteoclasts on periosteal surfaces, resulting in decreased periosteal circumference and cortical width, as well as increased fracture susceptibility (17, 18, 19, 20). The increased formation of osteoclasts has been attributed to enhanced formation of RANKL by ATRA, the biologically active metabolite of vitamin A (37). In the present investigation, we report the novel observation that ATRA increases osteoclast numbers by enhancing the number of macrophages in periosteal tissue, which serve as RANKL-sensitive osteoclast progenitor cells.

Newborn mouse calvarial cells, isolated by sequential collagenase digestions, are commonly used for studies of murine osteoblasts but have been shown to also contain mononuclear osteoclast progenitor cells (47, 48) with several characteristics of macrophages, including osteomacs (55, 56). This has also been demonstrated in a recent single-cell RNA-seq study (57), as well as in the present study. The cells isolated using collagenase digestions are released from both the outer and inner surfaces of the calvarial bones with no contact to bone marrow (48) and, therefore, we considered these cells as periosteal bone cells.

Treating the periosteal cells for 7 days with RANKL resulted in enhanced expression of osteoclastic genes and increased numbers of osteoclasts. This was not observed when periosteal cells were treated with ATRA alone, nor did ATRA affect the response to RANKL at day 7. However, when culture time was extended to 11 and 14 days, ATRA strongly potentiated the effect by RANKL on expression of osteoclastic genes and numbers of osteoclasts. When the periosteal cells were incubated on bone discs, osteoclastogenesis, as assessed by release of Trap5b, was robustly increased in cells treated with ATRA together with RANKL compared to the treatment with RANKL alone, resulting in increased formation of resorption pits and release of CTX. Similar to the observations made when cells were cultured on plastic dishes, the potentiation by ATRA on bone discs was observed after a significant delay. These observations suggested that ATRA does not increase osteoclast formation primarily by affecting the signaling downstream RANK but rather affects the composition of cells in the culture or the priming of RANKL-sensitive cells.

Studies have demonstrated that ATRA stimulates RANKL formation, osteoclastogenesis, and bone resorption in *ex vivo* organ cultures of neonatal mouse calvarial bones (37). Additionally, a high dose of vitamin A given to mice increases the number of periosteal osteoclast and mRNA expression of osteoclastic genes and *Tnfsf11* mRNA expression in cortical bone (19). These observations are in contrast to the findings of current study, in which ATRA did not stimulate the mRNA expression of osteoclastic genes, or the formation of mature osteoclasts in periosteal cell cultures on plastic dishes or on bone discs. This may be due to the fact that ATRA did not induce the mRNA expression of *Tnfsf11 (Rankl)* in the periosteal cell cultures. These observations indicate that the cells producing RANKL in response to ATRA *in vivo* and in *ex vivo* organ cultures are not isolated during enzymatic digestion of the calvarial bones, or that the RANKL-producing cells become unresponsive to ATRA after isolation. Osteocytes have been shown to be an important source for RANKL production *in vivo* and to be important for normal bone remodeling and for the response to unloading, oestrogen, glucocorticoids, and hyperparathyroidism (24, 25, 58, 59, 60). The periosteal cells used in the present experiments, did not express *Sost* mRNA, indicating the absence of osteocytes (data not shown). Further studies are needed to investigate the importance of osteocyte-derived RANKL for the *in vivo* effects of vitamin A.

Priming cells with ATRA in the current study resulted in a robustly enhanced response to RANKL, as demonstrated by significantly increased expression of osteoclastic genes and numbers of osteoclasts, compared with the response to RANKL when cells were precultured in control medium. This finding indicated that ATRA either increased the numbers of osteoclast progenitor cells, independently of the concomitant presence of RANKL, or that the sensitivity of individual cells to RANKL stimulation was increased.

The finding that ATRA treatment of the periosteal cells for 7 to 11 days progressively increased the mRNA expression of *Tnfrsf11a* and protein level of RANK provided an explanation for why priming with ATRA followed by RANKL treatment resulted in increased numbers of mature osteoclasts. However, it does not discriminate if this is due to increased RANK expression in individual osteoclast progenitors/macrophages or due to enhanced numbers of RANK-expressing progenitor cells. The fact that several genes expressed by macrophages such as *Csf1r*, *Irf8*, *Adgre1,* and *Itgam* also were upregulated by ATRA indicates that the number of macrophages/osteoclast progenitors, rather than their expression of RANK, is enhanced by ATRA. The upregulation of these genes was dependent on the concentration of ATRA with estimated half-maximal stimulation at ∼3 nM. Additional evidence for the hypothesis that ATRA increases the number of osteoclast progenitors/macrophages was provided by the observation that the numbers of cells stained for macrophage markers CD11b^+^F4/80^+^Gr1^-^ were robustly enhanced by ATRA.

Interestingly, the numbers of CD11b^+^F4/80^+^Gr1^−^ macrophages constituted ∼39% of total numbers of periosteal cells after treatment with ATRA for 11 days, compared to ∼15% in control cultures. The presence of macrophages in mouse periosteal cell isolations was also shown by Chang *et al.* (55) who found that ∼16% of the freshly isolated cells were F4/80^+^ macrophages, which increased during a 21-day culture as assessed by mRNA expression of *Adgre1* and immune histochemistry analyses of F4/80. These authors demonstrated the important role of the osteal tissue macrophages for osteoblast differentiation and bone noduli formation and, therefore, designated the cells as “osteomacs”. A large proportion of macrophages in mouse periosteal calvarial cells isolated by collagenase treatment was also demonstrated by a single-cell RNA-seq analysis showing that the proportion of cell expressing *Cd45*, *Adgre1*, *Cd68,* and *Csf1r* was ∼6% in freshly isolated cells but increased to ∼34% after a 12-day culture period in control medium (57). Ayturk *et al.* also reported that ∼2% of cells were CD45^+^/F4/80^+^ in freshly isolated cells, which increased to ∼51% after 12 days in culture in control medium. In agreement with these findings, we also observed that the cells expressing macrophage markers increased progressively in cells cultured in control medium. The capacity of these cells to differentiate into bone resorbing osteoclasts when stimulated by RANKL has been shown by Mohamad *et al.* (56) as well as in the present study.

In contrast to the stimulatory effect by ATRA in the periosteal cell cultures, ATRA did not affect the expression of genes associated with macrophages and osteoclast progenitor cells (*Adgre1*, *Csf1r*, *Tnfrsf11a*) in BMC cultures containing both macrophages and osteoblastic cells. In line with these findings, we have previously observed that ATRA does not affect proliferation of M-CSF–stimulated bone marrow macrophages (38) or M-CSF–stimulated CD14^+^ monocytes (43). Interestingly, it has been reported that calvarial periosteal macrophages and bone marrow macrophages are phenotypically different based upon mRNA and protein expression analyses (61), which may explain the different sensitivity to ATRA. In contrast to these observations, however, Hu *et al.* (44) have reported that ATRA increases the proliferation of human CD14^+^ monocytes and the monocytic mouse cell line RAW264.7 both in the absence and presence of RANKL. Although some controversy exists if ATRA can stimulate human monocytes, observations made by several laboratories show that ATRA is a robust inhibitor of RANKL-stimulated osteoclast formation using either primary bone marrow macrophages, the mouse monocytic cell line RAW264.7 or human peripheral blood monocytes (17, 38, 39, 40, 41, 42, 43, 44, 45). We believe that the inhibitory effect by ATRA on osteoclast differentiation of bone marrow macrophages/osteoclast progenitors explains why treatment with vitamin A decreases the numbers of endocortical osteoclasts *in vivo* (18, 19). How this inhibitory effect by ATRA relates to osteoclast formation on trabecular bone in mice treated with vitamin A is unclear, since in two studies demonstrating stimulation of periosteal osteoclasts and inhibition of endocortical osteoclasts by vitamin A treatment, no effect on the numbers of osteoclasts on trabecular bone was observed (18, 19).

The finding in the present study demonstrating that ATRA stimulates the number of periosteal macrophages, together with the previous finding demonstrating that ATRA increases RANKL formation (37), provides an explanation why excess of vitamin A increases periosteal osteoclast formation and decreases cortical bone mass in humans and mice (8, 9). It is, however, unclear as to why ATRA does not inhibit periosteal osteoclast progenitor cell differentiation or bone resorption in *ex vivo* cultured mouse calvarial bones (37), when such a strong inhibition of osteoclast differentiation is observed in the bone marrow osteoclast progenitors.

The important role of M-CSF for proliferation and survival of monocytes/macrophages is well recognized (53). It has also been shown that the numbers of F4/80^+^ osteomacs present in a tibial bone healing model can be enhanced by local injection of M-CSF, although it could not be concluded if this was due to proliferation, recruitment, or differentiation (62). We, therefore, assessed if the enhanced number of periosteal macrophages induced by ATRA was dependent on M-CSF. Surprisingly, ATRA did not increase the mRNA or protein expression of M-CSF, rather the opposite was found since ATRA significantly decreased M-CSF protein expression in the periosteal cells and the culture media. Furthermore, addition of M-CSF protein to the periosteal cells did not recapitulate the increased expression of macrophage/osteoclast progenitor genes induced by ATRA, nor did antiserum neutralizing M-CSF affect the response to ATRA. The lack of effect by M-CSF in calvarial periosteal macrophages has also been noted by Mohamad *et al.* (61) when comparing proliferation of these cells with the proliferative response to M-CSF in bone marrow macrophages. If this is due to high endogenous expression of M-CSF in the periosteal cultures, or if the explanation is that only a subgroup of macrophages in the periosteal cultures express *Csf1r* (56) remains an open question. The fact that macrophages in these two tissues exhibit several phenotypic differences suggests that also other mechanisms may contribute (61). Since it has been shown that IL-34 can activate M-CSF receptors and substitute for M-CSF during RANKL-stimulated osteoclastogenesis (23), we also assessed if ATRA enhanced IL-34 but found that ATRA robustly decreased both mRNA and protein expression of IL-34. Similar to M-CSF and in contrast to ATRA, addition of IL-34 protein did not affect the expression of macrophage/osteoclast progenitor genes in the periosteal cell cultures.

In one study, it has been shown that high vitamin A diet given to mice cause decreased bone mass and thickness also in calvarial bones, an effect associated with increased number of osteoclasts (54). In the skull bones, the increase of osteoclasts is observed on endocranial surfaces while in long bones the increase of osteoclasts is seen on periosteal surfaces. Both are surfaces not in contact with the bone marrow compartment. The lack of effect on osteoclast number on the pericranial surface *in vivo* indicates that the RANKL-expressing cells responding to ATRA are present in different compartment in calvarial bones and long bones. Because enhanced osteoclastogenesis can be seen in both calvarial and long bones in response to vitamin A and since the periosteal cells used in the present study are isolated from both peri- and endocranial surfaces, our observations are likely representative for long bones.

To demonstrate that vitamin A enhances macrophage/osteoclast progenitor cells in long bones *in vivo*, we treated mice with ATRA through oral gavage for a short period of time to avoid that all macrophages/osteoclast progenitors were differentiated to mature osteoclasts. We could demonstrate that hypervitaminosis A increased the expression of the macrophage/osteoclast progenitor genes *Csf1r*, *Tnfrsf11a, Itgam,* and *Cd68* in the periosteum of tibia. This observation indicates that ATRA can increase the number of macrophage/osteoclast progenitor cells also in long bones *in vivo*, although formal proof must await studies using FACS analyses and/or morphological techniques.

In the present study, we show that macrophages are present in mouse calvarial periosteum and that the vitamin A metabolite ATRA stimulates the number of macrophages, resulting in enhanced numbers of osteoclast progenitor cells. This finding demonstrates a novel mechanism by which vitamin A enhances osteoclast formation specifically on bone surfaces not in contact with the bone marrow compartment. It remains to elucidate, however, to what extent enhanced proliferation and/or decreased apoptosis contribute to the effect by ATRA. It also remains to be elucidated if the stimulatory effect by ATRA is mediated by receptors expressed in macrophages of if the effect is indirect due to stimulation of osteoblasts releasing macrophage stimulatory factor(s).

## Experimental procedures

### Primary periosteal bone cell cultures

Primary calvarial periosteal bone cells from 3 to 5 days old C57BL/6N mice were isolated by sequential enzymatic digestion (48, 63). Ethical permit for the use of cells from mice was approved by the Ethical Committee for Animal Research in Gothenburg and the care of the animals was in accordance with relevant guidelines and regulations. Approximately 10 to 15 dissected calvariae were washed in PBS and incubated in 5 ml 4 mM EDTA in PBS at 37 °C, 600 rpm rotation table for two sequential 10 min digestions. Thereafter, the calvarias were incubated with 180 U/ml collagenase type II (cat no LS004176, Worthington, BioNordika) in PBS for seven sequential 10 min, 37 °C, 600 rpm, 5 ml digestions. Collagenase fractions were pooled and periosteal bone cells were cultured in complete alpha minimum essential medium (α-MEM) (cat no 22561-021, Gibco, Thermo Fisher Scientific) supplemented with 10% heat inactivated fetal bovine serum (cat no F7524, Sigma), 2 mM GlutaMAX (cat no 35050-038, Gibco), 50 μg/ml gentamicin (cat no 15750-037, Gibco), 100 U/ml penicillin, and 100 μg/ml streptomycin (cat no 15140-148, Gibco) for 3 to 5 days prior to the experiments. At the start of experiments, cells were seeded at 20.000 cells/cm^2^ in osteogenic media (complete α-MEM medium supplemented with 2 mM β-glycerophophate, cat no G9422, Sigma, and 0.2 mM L-ascorbic acid 2-phosphate sesquimagnesium salt hydrate, cat no A8960, Sigma) with or without 0.01 to 100 nM ATRA (cat no R2625, Sigma), 4 ng/ml RANKL (cat no 462-TEC-010, R&D), 30 ng/ml M-CSF (cat no 416-ML/CF, R&D), 4 μg/ml anti-M-CSF (cat no MA5-23863, Invitrogen), 100 ng/ml IL-34 (cat no 5195-ML-010/CF, R&D), or 100 ng/ml OSM (cat no 495-MO-025/CF, R&D). Culture media was replenished every 3 to 4 days, and experiments were terminated at indicated time points. In most experiments, the cells were cultured for a comparatively longer period of time (up to 11–14 days) than the other cell culture used for osteoclastogenesis experiments. The reason is the relatively low numbers of macrophages/osteoclast progenitors present at the start of experiments, which need to be expanded until osteoclast formation is induced. For all experiments, except for bone resorption and protein/FACS analyses, 48-well plates were used. Bone resorption and protein/FACS analyses were performed in 96- and 6-well plates, respectively.

The isolated bone cells were harvested from both pericranial and endocranial surfaces with no contact to bone marrow (48). Since the cells were isolated from newborn mice, both bone forming osteoblasts and bone resorbing osteoclasts were present at both surfaces and consequently the cells isolated contain both osteoblasts and osteoclast progenitor cells/macrophages (47, 48). We consider both pericranial and endocranial tissues being of periosteal nature and the isolated cells are therefore called periosteal bone cells.

### Analyses of osteoclast formation and bone resorption

To visualize TRAP^+^ osteoclasts, cells were fixed and stained for TRAP using a commercial staining kit (cat no 387A, Sigma-Aldrich). TRAP-positive cells with two or more nuclei were defined as MuOCLs.

To study bone resorption, periosteal bone cells were cultured on bovine bone discs (cat no TDT-1BON1000-96, IDS Diagnostics) in 96-well plates as described above. TRAP5b as a measure of osteoclastogenesis was analyzed in the culture medium using commercial TRACP 5b ELISA (cat no SB-TR103, IDS Diagnostic). Resorption pits were visualized by reflective light microscopy and degree of resorption was analyzed by measuring the release of CTX-I in bone cell culture supernatants using commercial ELISA (cat no AC-06F1, IDS Diagnostics).

Number of TRAP-positive cells, MuOCL, and percent resorption pits surface per bone surface were analyzed using a Nikon Eclipse 80i microscope and the Osteomeasure7 v4.3.0.1 software (www.osteometrics.com).

### BMC cultures

BMC were flushed from femur and tibia of 10- to 12-week-old male C57BL/6N mice and washed once in complete α-MEM. BMC were spot-seeded at 750.000 cells in 30 μl complete α-MEM in 48-well plates for 5 min, after which 250 μl complete α-MEM was added. The following day, all media were removed and replaced by osteogenic media with or without 100 nM ATRA. Half of the media volume was substituted with fresh media on day 3 and 7. Cells were stained with commercial kits for alkaline phosphatase (cat no 85L2-1KT, Sigma) and TRAP (cat no 387A, Sigma-Aldrich) or harvested for gene expression analyses on day 7 and 11.

### Gene expression analyses

For gene expression analysis, cells were lysed in RNeasy Lysis buffer (Qiagen) with 1% 2-mercaptoethanol (cat no M6250, Sigma-Aldrich), followed by RNA purification using an RNAeasy Micro (Qiagen) kit. Single-strand complementary DNA (cDNA) was synthesized using a high-capacity cDNA reverse transcription kit (cat no 4374967, Applied Biosystems). Quantitative real time PCR analyses were performed by using predesigned Taqman Assays and Taqman Fast Advance Master Mix and the StepOnePlus Real-Time PCR system (Applied Biosystems). The following predesigned real-time PCR assays from Applied Biosystems were used for gene expression analysis: *Acp5* (*Trap*; Mm00475698_m1), *Ctsk* (Mm00484036_m1), *Calcr* (Mm00432252_m1), *Tnfrsf11a* (*Rank*; Mm00437135_m1), *Tnfsf11 (Rankl*; Mm00441908_m1), *Tnfrsf11b* (*Opg*; Mm00435452_m1), *Csf1* (*Mcsf*; Mm00432686_m1), *Csf1r* (*M-csf-r;* Mm01266652_m1), *Nfatc1* (Mm00479445_m1), *Adgre1* (*F4/80*; Mm00802529_m1), *Itgam* (*CD11b*; Mm00434455_m1), *Irf8* (Mm00492567_m1), *Cd68* (Mm03047343_m1), and *Il34* (Mm01243248_m1). House-keeping gene 18S (cat no 4310893E) was used as an endogenous control. Data were calculated using the 2^−ΔΔCt^ method and shown as fold changes relative to the group having the lowest detectable levels of the transcript at the first time point. For all analyzed genes except *Calcr* the group without treatment had the lowest expression and expression was expressed as fold *versus* the control without treatment. *Calcr* is highly osteoclast specific, and no expression is seen without the presence of RANKL. For *Calcr*, the expression was calculated as fold difference compared to the RANKL treated group. Average Ct values of respective gene and 18S in the reference group for each figure are shown in Fig. S2.

### Protein preparation and analysis of RANK

Primary calvarial periosteal cells were isolated and cultured with or without ATRA as described above in 6-well plates. After 11 days, protein lysates were prepared by washing the cells once with cold PBS, followed by lysis in radioimmunoprecipitation assay buffer, Sigma, R0278) with protease inhibitor (complete Mini EDTA-free; RocheDiagnostics, 5892970001) and phosphatase inhibitor (PhosSTOP; Roche Diagnostics, 04906845001). Lysates were transferred to tubes, centrifuged for 10 min at 11.000*g*, +4 °C, and the supernatant was used for protein analysis. Protein concentration was quantified using the detergent compatible protein assay kit (Bio-Rad, 500-0112). Levels of RANK were analyzed by capillary-based electrophoresis and immunodetection using the Jess ProteinSimple system and the Compass Software (Protein Simple, www.bio-techne.com) as described by the manufacturer. Protein lysates were analyzed with anti-RANK (H-7) (sc-374360, Santa Cruz Biotechnolog Inc, dilution 1:10) together with the protein normalization assay module (ProteinSimple, AM-PN01) and the anti-mouse detection module (ProteinSimple, DM-001) in 12 to 230 kDa capillary separation cassettes (ProteinSimple, SM-W004). RANK was visually presented in the lane view of the Compass Software and quantified by normalization to total protein levels according to the Simple Western protocol.

### Cytokine measurements in culture media

M-CSF/CSF1 and IL-34 levels in cell lysates and culture media were measured using commercial ELISAs (cat no MMC00 and M3400, R&D). Cell lysates were prepared from cell cultures in 48-well plates by removing the culture media, adding 75 μl PBS to the wells and freezing at −80 °C. After thawing, 75 μl cell lysis buffer 2 (cat no 895347, R&D) was added and the cells were lysed at room temperature with gentle agitation for 30 min. Lysates were centrifuged at 10.000*g* for 5 min and supernatants were used in ELISA.

### Flow cytometry

Periosteal cells were cultured in 6-well plates or 10 cm dishes in osteogenic media with or without ATRA for 11 days. Cells were detached with trypsin, filtered through a 100 μm cell strainer, pelleted, and resuspended in FACS buffer (PBS supplemented with 10% fetal bovine serum, cat no F7524, Sigma). Cell concentration was determined using Nucleocassettes and Nucleocounter NC-100 (Chemometec). The cells were incubated with Fc block (Clone 2.4G2, Becton Dickinson (BD)) and stained with Violet 450–conjugated anti-CD11b (Clone M1/70, BD), allophycocyanin (APC)/cyanine7-conjugated anti-F4/80 (Clone BM8, BioLegend), and PerCP/Cyanine5.5-conjugated anti-Ly-6G/ly-6C (Gr-1) (Clone RB6-8C5, BioLegend). Fluorescence minus one and unstained samples were used as controls. The cells were acquired in FACSVerse (BD) and FlowJo software version 10.8.0 (Tree Star, www.flowjo.com) was used for data analysis.

### Treatment of adult mice with ATRA

Animal experiments were approved by the Ethical Committee for Animal Research in Gothenburg and the care of the animals was in accordance with relevant guidelines and regulations. Pellet diet (1324 maintenance diet) and water were available *ad libitum*. Nine-week-old female C57BL/6N mice from Taconic were treated by oral gavage with ATRA (Tretinoin, cat no PHR1187, Sigma-Aldrich, 110 mg/kg/day) or vehicle (corn oil, cat no C8267, Sigma-Aldrich) once per day for 3 days and terminated on day 4. The mice were euthanized using Ketador (Richter Pharma) mixed with Dexdomitor (Orion Pharma), followed by exsanguination and cervical dislocation. Tibia were dissected and cleaned from soft tissue without disrupting the periosteum and marrow cavity and stored in RNAprotect (cat no 76106, Qiagen) at −80 °C until RNA preparation. For periosteal RNA preparation, tibias were put in TRIzol and vortexed for 1 min before chloroform extraction followed by the Qiagen RNesay mini kit protocol. Single-strand cDNA synthesis and gene expression was analyzed as above.

### Statistical analysis

GraphPad Prism statistical software (GraphPad Software, Inc, www.graphpad.com) was used for all analyses. Student’s unpaired, two-tailed *t* test was used to compare two groups. When three or more treatments were included, one-way ANOVA followed by Dunnett’s multiple comparison test was used to compare each treatment *versus* control, and Tukey’s multiple comparison test to compare all treatments to each other. Two-way ANOVA followed by Sidak’s multiple comparison test was used to compare the effect of RANKL in cells with and without ATRA. Figures are displayed as scatter plots of individual values with mean ± SD with *p* <0.05 considered statistically significant. All cell culture experiments were performed with four technical replicates (wells) per group and repeated at least twice with comparable results. Animal experiments were performed with ten mice per group.

## Data availability

The datasets generated during and/or analyzed during the current study are available from the corresponding author on reasonable request.

## Supporting information

This article contains supporting information.

## Conflict of interest

The authors declare that they have no conflicts of interest with the contents of this article.
