# Supplementary material for: Vitamin A enhanced periosteal osteoclastogenesis is associated with increased number of tissue-derived macrophages/osteoclast progenitors
Source: J Biol Chem. 2024 Apr 23;300(6):107308. doi: 10.1016/j.jbc.2024.107308 (PMC11163173; doi:10.1016/j.jbc.2024.107308)
Supplement: Supporting Figures S1 and S2 [file mmc1.pdf]

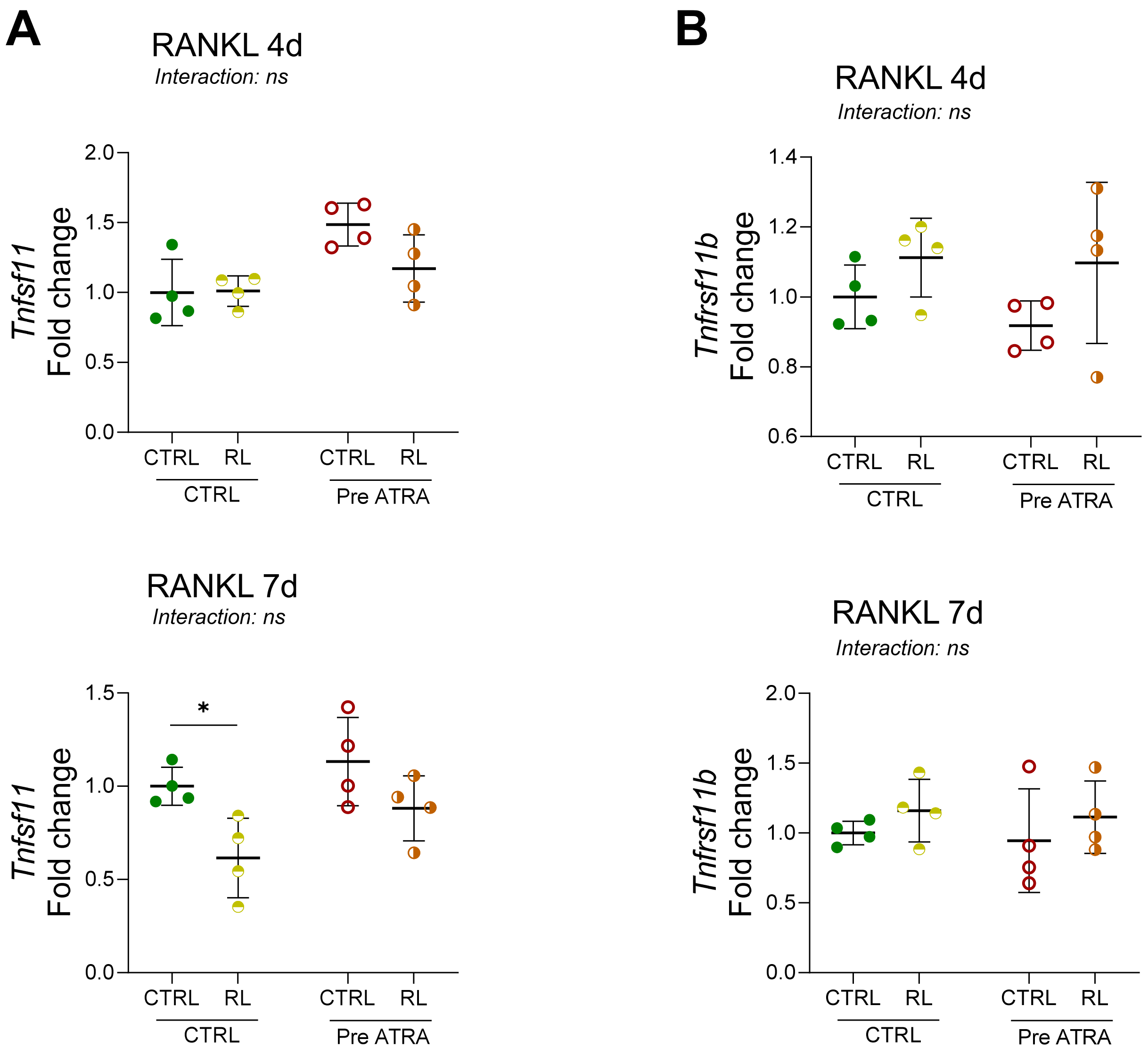

Supporting information 1: Pre-treatment with ATRA did not alter the expression of *Tnfsf11* (*Rankl*) or *Tnfrsf11b* (*Opg*). Periosteal bone cell cultures were pre-incubated with or without ATRA (100 nM) for 7 days. Thereafter, ATRA or control media was replaced by control or RANKL (4 ng/ml) containing media. Gene expression of *Tnfsf11* (*Rankl*) (A) and *Tnfrsf11b* (*Opg*) (B) was analysed 4 and 7 days after the addition of RANKL. Figures are displayed as scatter plots of replicate wells with mean ± SD. 2-way ANOVA followed by Sidak's multiple comparison test for the effect of RANKL; * $P < 0.05$ vs. CTRL; interaction non-significant (ns) for the RANKL response in cells pre-treated in CTRL vs ATRA containing media.

Supporting information 2: Table of quantitative PCR average Ct values of reference group in each figure.

Figure number	Gene of interest	Reference group	Average Ct 18S	Average Ct Gene of interest
1B	*Acp5*	CTRL day 4	9,3	28,7
1C	*Ctsk*	CTRL day 4	9,9	23,2
1D	*Tnfsf11*	CTRL day 4	10,3	29,3
2A	*Acp5*	CTRL day 7	17,7	30,2
2B	*Ctsk*	CTRL day 7	14,0	26,3
2C	*Calcr*	RL+ATRA day 7	14,2	35,9
3A RL 4 days	*Acp5*	CTRL+CTRL	12,6	29,9
3A RL 7 days	*Acp5*	CTRL+CTRL	12,5	30,0
3B RL 4 days	*Ctsk*	CTRL+CTRL	11,0	25,5
3B RL 7 days	*Ctsk*	CTRL+CTRL	11,1	25,4
3C RL 4 days	*Calcr*	CTRL+RL	11,4	34,0
3C RL 7 days	*Calcr*	CTRL+RL	11,4	35,8
3D RL 4 days	*Nfatc1*	CTRL+CTRL	11,6	30,3
3D RL 7 days	*Nfatc1*	CTRL+CTRL	11,9	29,6
4A	*Csf1r*	CTRL day 7	14,9	29,0
4B	*Tnfrsf11a*	CTRL day 7	13,2	35,3
4C	*Irf8*	CTRL day 7	13,1	35,9
4D	*Adgre1*	CTRL day 7	13,5	33,2
4E	*Itgam*	CTRL day 7	13,2	30,3
4F	*Adgre1*	0 nM	13,2	27,4
4G	*Csf1r*	0 nM	11,3	27,2
6A	*Csf1r*	CTRL day 7	11,0	23,3
6B	*Adgre1*	CTRL day 7	12,3	25,4
6C	*Tnfrsf11a*	CTRL day 7	8,9	27,8
7A	*Csf1r*	CTRL day 3	11,4	25,6
7D	*Adgre1*	CTRL	12,1	27,3
7D	*Csf1r*	CTRL	12,3	27,5
7D	*Tnfrsf11a*	CTRL	11,4	32,3
7E	*Adgre1*	CTRL+CTRL	13,3	27,6
7F	*Csf1r*	CTRL+CTRL	10,0	27,5
7G	*Tnfrsf11a*	CTRL+CTRL	13,2	32,7
7H	*Il34*	CTRL day 3	11,0	31,3
7K	*Adgre1*	CTRL	12,9	29,9
7K	*Csf1r*	CTRL	11,5	29,1
7K	*Tnfrsf11a*	CTRL	12,0	34,2
8A	*Csf1r*	CTRL	13,2	26,7
8B	*Adgre1*	CTRL	13,3	28,9
8C	*Tnfrsf11a*	CTRL	13,2	31,5
8D	*Itgam*	CTRL	12,9	28,7
8E	*Irf8*	CTRL	13,2	31,1
8F	*Cd68*	CTRL	12,8	26,4
S2A RL 4 days	*Tnfsf11*	CTRL+CTRL	11,4	31,0
S2A RL 7 days	*Tnfsf11*	CTRL+CTRL	11,7	30,9
S2B RL 4 days	*Tnfrsf11b*	CTRL+CTRL	11,3	29,7
S2B RL 7 days	*Tnfrsf11b*	CTRL+CTRL	11,6	29,8
